# FTO5/470: Critical Incident Monitoring in Emergency Medicine Web-based System (CRIME-base): Current evidence on incident reporting and its impact to quality of care

**DOI:** 10.2196/jmir.1.suppl1.e30

**Published:** 1999-09-19

**Authors:** TN Arvanitis, J Ryan

**Affiliations:** ^1^The University of BirminghamEdgbaston, BirminghamUK

**Keywords:** CRIME-base Brighton, Critical Incident Monitoring, Risk Management

## Abstract

**Introduction:**

A critical incident is "any event which is inconsistent with routine hospital practice or with the quality of patient care and which has or could have had a demonstrably adverse outcome for a particular patient" (J.A. Williamson, Critical Incident Reporting in Anaesthesia. *Anaesthetic Intensive Care,* 1988: 16: 101-103). Such "negative incidents" may arise as a result of a variety of reasons during routine hospital practice. Incident monitoring and reporting has been identified as a form of clinical action research, that allows for the elicitation of appropriate information towards the planning of risk management in hospital practice. In particular, incident monitoring in emergency medicine is directly related to activities such as risk management planning, "best practice" guideline and protocol design, while it informs intra-departmental educational activities. Incident monitoring in Accident & Emergency (A&E) hospital-based Department has been traditionally performed through a paper-based reporting system. The major disadvantages of such an approach are that 1) paper reporting is time consuming 2) the anonymity of the reporter might not be preserved 3) most reports are submitted from senior staff and 4) reports are frequently event-centric rather than patient-focused.

**Methods:**

We have developed a simple Web-based reporting approach to facilitate the electronic submission of critical incident reports in A&E. An on-line HTML form provides a simple, easy-to-use interface for submission of critical incident reports that are stored in an Microsoft Access Database Management System. The reported information is stored in relational records with elements that identify the type and experience of personnel involved in the incident, patient and clinical history details, location where the incident occurred, triage category, the nature of complaint (medical/surgical). Furthermore, the classification of patient outcome is reported in addition with some suggestion from the reporter on the factors contributing towards the incident, corrective actions taken and recommendations on future prevention of similar events. In this paper, we present current evidence of critical evidence in an A&E Department and we discuss the implications of such evidence to the planning of risk management, and hence the impact of such an approach to improving the quality of patient care.

**Results:**

Over the period of two years, we have run a pilot study of incident reporting at an A&E Department. This study has ensured anonymity in reporting. Patient-centric critical incident data has been gathered. We present data that relates to nature of the problem (medical or surgical) to triage category, drug error and type of complaint. This information is correlated to the reasons of incident occurrence. Our study showed that 41% of incidents occurred because of personnel lack of experience with the clinical problem, 27% due to carelessness in patient handling, 9% due to inappropriate hand-over and, 8% due to inadequate history/examination, 5 % to work-overload, 5% due to communication difficulties, and 5%to other reasons. A high correlation between the number of re-occurring events and the level of experience of personnel (identified as personnel grade and length of time in service) has been identified.

**Discussion and Conclusion:**

The evidence of our study has identify the following benefits of the Web-based critical incident monitoring: 1) allowed us to re-address induction courses for junior personnel 2) helped us to focus teaching to areas of clinical need 3) identified the needs for more appropriate Risk Management 4) offered the opportunity to share sensitive information and 5) reduced the burden of the previously use paper-based approach.

